# Non-Muscle Myosin II Regulates Neuronal Actin Dynamics by Interacting with Guanine Nucleotide Exchange Factors

**DOI:** 10.1371/journal.pone.0095212

**Published:** 2014-04-21

**Authors:** Eun-Young Shin, Chan-Soo Lee, Cheong-Yong Yun, So-Yoon Won, Hyong-Kyu Kim, Yong Hee Lee, Sahng-June Kwak, Eung-Gook Kim

**Affiliations:** 1 Department of Biochemistry, College of Medicine and Medical Research Institute, Chungbuk National University, Cheongju, Chungbuk, Korea; 2 Division of Hazardous Substances Analysis, Daejeon Regional Food and Drug Administration, Daejeon, Chungnam, Korea; 3 Department of Microbiology, College of Medicine and Medical Research Institute, Chungbuk National University, Cheongju, Chungbuk, Korea; 4 Department of Biochemistry, College of Medicine, Dankook University, Cheonan, Chungnam, Korea; UPR 3212 CNRS -Université de Strasbourg, France

## Abstract

**Background:**

Non-muscle myosin II (NM II) regulates a wide range of cellular functions, including neuronal differentiation, which requires precise spatio-temporal activation of Rho GTPases. The molecular mechanism underlying the NM II-mediated activation of Rho GTPases is poorly understood. The present study explored the possibility that NM II regulates neuronal differentiation, particularly morphological changes in growth cones and the distal axon, through guanine nucleotide exchange factors (GEFs) of the Dbl family.

**Principal Findings:**

NM II colocalized with GEFs, such as βPIX, kalirin and intersectin, in growth cones. Inactivation of NM II by blebbistatin (BBS) led to the increased formation of short and thick filopodial actin structures at the periphery of growth cones. In line with these observations, FRET analysis revealed enhanced Cdc42 activity in BBS-treated growth cones. BBS treatment also induced aberrant targeting of various GEFs to the distal axon where GEFs were seldom observed under physiological conditions. As a result, numerous protrusions and branches were generated on the shaft of the distal axon. The disruption of the NM II–GEF interactions by overexpression of the DH domains of βPIX or Tiam1, or by βPIX depletion with specific siRNAs inhibited growth cone formation and induced slender axons concomitant with multiple branches in cultured hippocampal neurons. Finally, stimulation with nerve growth factor induced transient dissociation of the NM II–GEF complex, which was closely correlated with the kinetics of Cdc42 and Rac1 activation.

**Conclusion:**

Our results suggest that NM II maintains proper morphology of neuronal growth cones and the distal axon by regulating actin dynamics through the GEF–Rho GTPase signaling pathway.

## Introduction

The neuronal growth cone is a specialized motile apparatus, which is located at the tips of extending neurites (axons or dendrites). The dynamic assembly and remodeling of filamentous actin (F-actin) determine growth cone behaviors, which include changes in shape and motility, as well as turning towards external guidance cues. Two distinct types of F-actin–based structures, lamellipodia, which are composed of a fine actin meshwork, and filopodia, which contain actin bundles, appear at the periphery (P-domain) of growth cones. The assembly of new actin at the leading edge induces local protrusion of lamellipodia and filopodia, and thus promotes motility or turning responses of growth cones [1]. This assembled F-actin undergoes retrograde actin flow and depolymerization at the pointed end near the actin arc [2]–[5]. These processes are equally important in the modulation of growth cone behavior [6].

Guanine nucleotide exchange factors (GEFs) of the Dbl family are upstream activators of the Rho GTPases Rac1, Cdc42 and RhoA, which induce the formation of lamellipodia, filopodia and stress fibers, respectively [7]. Rho GTPases are involved in diverse cellular processes, including cell migration and adhesion, mitosis, synapse formation and neurite outgrowth [8]. Therefore, the activity and localization of Rho GTPases are under tight spatio-temporal regulation in these processes. In neuronal growth cones, Rho GTPases regulate actin dynamics, which is required for maintaining growth cone morphology and motility [9]. Although the downstream effectors of Rho GTPases for actin polymerization and depolymerization have been intensively studied, the mechanism for targeting their upstream GEFs to the leading edge or to the filopodial tips of growth cones is largely unknown.

Neuronal growth cones contain various types of myosins, including classes I, II, V and VI. Among them, non-muscle myosin II (NM II) has been intensively studied in association with growth cone behavior. NM II belongs to the conventional myosin II subfamily and consists of pairs of essential light chains, regulatory light chains and myosin heavy chains (MHCs). Three MHC isoforms of NM II, IIA, IIB and IIC, have been identified to date. In growth cones, NM IIB is primarily located in the transition zone (T-zone) and is dispersed throughout the P-domain, whereas NM IIA is primarily concentrated in the central domain (C-domain) [10]. The contractile force of NM II is responsible for retrograde actin flow [11], [12]. Furthermore, Medrios and coworkers [13] demonstrated that NM II is involved in severing actin bundles at the actin arc, which is followed by actin depolymerization. Inhibition of NM II ATPase activity by a specific inhibitor, blebbistatin [14], makes the leading edge more dynamic by increasing the frequency of protrusions and retractions, induces frequent changes in the growth cone shape and reduces directed motility [13]. In fibroblasts, specific depletion of NM IIA or IIB resulted in a similar (2–3-fold) increase in the rate of protrusion [15]. These results suggest that the loss of NM II contractility by direct inhibition of its ATPase activity inversely correlates with actin dynamics. We previously proposed a potential mechanism to explain this inverse correlation [16]. When NM II is active, it inhibits the activity of Dbl family GEFs. Conversely, when NM II is inactive (for instance, due to blebbistatin treatment), GEFs are released and partially activated, which activates Rac1 and promotes actin dynamics in fibroblasts.

Because NM II is involved in the protrusion of the leading edge via its interaction with GEFs, it may control the shape of the growth cone and the distal axon by a similar mechanism. Therefore, we hypothesize that NM II may recruit GEFs of the Dbl family near the peripheral actin and the central domain, and suppress their localization on the shaft of the distal axon. Here we demonstrate that NM II modulates the localization and activity of Dbl family GEFs and influences actin dynamics at the leading edge of growth cones and in the distal axon. Our data suggest a novel role for NM II and GEFs in regulation of the growth cone shape and actin dynamics.

## Materials and Methods

### Materials

Anti-NM IIB (N-17), myosin light chain (MLC) (E-2), phospho-MLC (S18/T19), kalirin (C-20), intersectin (ITSN) (H-16) and GFP antibodies were purchased from Santa Cruz Biotechnology (Santa Cruz, CA, USA). Anti-phospho-threonine, and Alexa Fluor 488 and 594–conjugated secondary antibodies were purchased from Invitrogen (Carlsbad, CA, USA). The anti-βPIX monoclonal antibody was raised against amino acids 439–648 of βPIX [17]. Blebbistatin (BBS) was obtained from Tocris Bioscience (Minneapolis, MN, USA). Dulbecco's Modified Eagle's Medium (DMEM), fetal bovine serum (FBS), Lipofectamine 2000, neurobasal media and stealth siRNAs for βPIX (βPIX-1: 5′- AAACUUUGCUCUUACUACCAGUUGA, βPIX-2: 5′-GGAGGAUUAUCAUCCUGAUAGACAA, βPIX-3: 5′-ACGACUGCCAUCAACAAGAGCUAUU) were purchased from Invitrogen. TRITC- and Alexa Fluor 488–conjugated phalloidin, and Duolink *In Situ* Detection Reagent Kit were obtained from Sigma-Aldrich (St. Louis, MO, USA). Raichu-Cdc42 and Rac1 probes for fluorescence resonance energy transfer (FRET) analysis were kindly provided by Dr. Matsuda Michiyuki (Osaka University, Osaka, Japan).

### Plasmid constructs

GFP-tagged constructs, including Cdc42-WT, Cdc42V12, dominant negative (DN) Cdc42N17 and RhoAV14, were cloned into pEGFP-C2 (Takara Bio Inc., Shiga, Japan). WT-PAK1 and DN PAK1 (H83/86L, K299R) cDNAs were cloned into pCMV-myc (Takara Bio Inc.). DH domain constructs were cloned into pEGFP-C2 using polymerase chain reaction (PCR). The inserts corresponded to amino acids 100–276 of wild-type (DH^WT^) or L238R/L239R (DH^mt^) βPIX [18] or to amino acids 1048–1239 of wild-type (DH^WT^) or L1048R/L1049S (DH^mt^) Tiam1.

### Cell cultures

Fetal rats were obtained from timed-pregnancy Sprague-Dawley (SD) rats at a gestational age of 18 days (E18). Rats were euthanized by CO_2_ exposure followed by cervical dislocation. Embryos with intact amniotic sacs were removed from the dam. Primary hippocampal neuron cells were extracted and isolated based on a previously described method [19]. Briefly, hippocampi were dissected, incubated with trypsin in HBSS (Hank's' Balanced Salt Solution) without Ca^2+^ and Mg^2+^ (pH 7.4) for 30 min at 37°C and dissociated by pipetting. Neurons were plated onto glass coverslips coated with 50 µg/ml poly-d-lysine in borate buffer (pH 8.3) at a density of 2×10^5^ cells per coverslip, and incubated at 37°C and 5% CO_2_ in a humidified incubator_._ After the neurons had attached to the substrate, the medium (neurobasal medium containing B27 supplement, 0.5 mM glutamine, 25 µM glutamate, and 100 U/ml penicillin/streptomycin) was changed to fresh growth medium without glutamate. PC12 cells were cultured in DMEM supplemented with 10% FBS and 100 U/ml penicillin/streptomycin at 37°C and 5% CO_2_ in a humidified incubator.

### Immunocytochemistry

Cells were fixed for 15 min with 3.7% paraformaldehyde in phosphate buffered saline (PBS), permeabilized for 5 min with 0.2% Triton X-100, and blocked for 30 min at 25°C with 2% bovine serum albumin (BSA) in PBS. Cells were incubated with primary antibodies for 1 h at 25°C, followed by incubation with secondary Alexa Fluor 488- or 594–conjugated antibodies for 1 h at 25°C. To visualize actin, cells were stained with TRITC-conjugated phalloidin for 30 min at 25°C. After staining, coverslips were mounted onto a glass slide, and observed and photographed with an Olympus IX81-ZDC inverted microscope (Olympus Optical Co., Tokyo, Japan) equipped with a cooled CCD camera, Cascade 512B (Photometrics, Tucson, AZ, USA). MetaMorph software version 7.1.7 (Molecular Devices, Downingtown, PA, USA) was used to control the imaging system.

### Immunoprecipitation and immunoblotting

Cells were lysed with lysis buffer (50 mM HEPES, pH 7.5, 150 mM NaCl, 10% glycerol, 1% Triton X-100, 500 µM EDTA, 200 µM sodium pyruvate, 50 mM β-glycero-phosphate), and proteins were immunoprecipitated from cell lysates with primary antibodies at 4°C for 18 h. Immunoprecipitates were collected by adding protein G agarose and washed five times with lysis buffer. Samples were fractionated by 8–10% SDS-PAGE and transferred to a polyvinylidene fluoride membrane in a Tris-glycine-methanol buffer (25 mM Tris base, 200 mM glycine and 20% methanol). Membranes were blocked with 3% BSA in Tris-buffered saline (TBS-T; 50 mM Tris, 150 mM NaCl, 0.1% Tween-20) for 30 min, incubated with the primary antibodies for 1 h at room temperature (RT), and washed three times with TBS-T. Membranes were then incubated with secondary horseradish peroxidase–conjugated antibody for 1 h at RT and washed with TBS-T three times. Signals were detected using an enhanced chemiluminescence reagent.

### FRET analysis

Cells were plated on 18-mm glass coverslips coated with poly-l-lysine. One day after plating, cells were transfected with the Raichu Cdc42 plasmid using Lipofectamine 2000 (Invitrogen) according to the manufacturer's protocol. Twelve hours after transfection, cells were treated with or without 50 µM BBS for 10 min and fixed in 3.7% paraformaldehyde/PBS for 10 min at RT. For FRET, cells were imaged with an Olympus IX81-ZDC inverted microscope equipped with a cooled CCD camera (Cascade 512B); the imaging system was controlled by the MetaMorph software version 7.1.7. With excitation at 433 nm, increases and decreases in FRET were observed at the emission peaks of 530 nm and 475 nm, respectively, using a 440AF21 excitation filter, a 455DRLP dichroic mirror and two emission filters (480AF30 for CFP and 535AF26 for FRET) (Omega Optical, Brattleboro, VT). Cells were illuminated with a 75-W Xenon lamp through a 12% ND filter and a 40X UPlanSApo objective. The exposure time for 2×2 binning was 200 ms to obtain images of CFP and FRET. After the background was subtracted from the captured images, FRET/CFP ratio images were generated by dividing the FRET image by the CFP image using the MetaMorph software version 7.1.7; these images were used to represent FRET efficiency.

### GST-PBD pulldown assay

To measure the activation of Cdc42/Rac1, GST-PBD (p21-binding domain of PAK1, amino acids 67–150) was expressed in *E. coli* (DH5α) and purified by glutathione-Sepharose affinity chromatography [20]. Lysates were incubated with the purified immobilized GST-PBD protein at 4°C for 2 h in binding buffer (25 mM Tris HCl, pH 7.5, 1 mM DTT, 30 mM MgCl_2_, 40 mM NaCl, 0.5% NP-40) and washed five times with lysis buffer. Beads were boiled in an SDS buffer for 5 min, resolved by 12% SDS-PAGE and transferred to a PVDF membrane. Membranes were immunoblotted with anti-Cdc42, Rac1 or GST antibody.

### *In situ* proximity ligation assay

To observe the direct interaction between GEF (βPIX and kalirin) and NM IIB in growth cones, a proximity ligation assay (PLA) was performed using the Duolink *In Situ* Detection Reagents Red. Cells were plated on 18-mm glass coverslips coated with poly-l-lysine, fixed with 3.7% paraformaldehyde in PBS, permeabilized for 5 min with 0.2% Triton X-100 and blocked with 3% BSA in PBS for 60 min. Cells were then incubated with antibodies against βPIX (or kalirin) and NM IIB (1∶200 each) in 3% BSA overnight at 4°C, followed by incubation with corresponding secondary antibodies conjugated with PLA probes for 1 h at 37°C. Ligation and amplification reactions were performed according to the manufacturer's instruction. The solution for the latter reaction contains a fluorophore (λex = 594 nm and λem = 624 nm) for visualization of ligated molecules by fluorescence microscopy. Finally, F-actin was stained with Alexa Fluor 488–conjugated phalloidin. Images were photographed and analyzed as described above for immunocytochemistry.

### Transient transfection

Transient transfection was performed with Lipofectamine 2000. Briefly, 100 nM siRNA or 5 µg of DNA were diluted with 5 µl of Lipofectamine 2000 in OPTI-MEM and added to culture dishes according to the manufacturer's instructions. After 24 h or 72 h, transfected cells were analyzed by immunoblotting with appropriate antibodies or monitored under a fluorescence microscope (Olympus, IX81).

### Ethics statement

We obtained a permission for animal experiments described in this study from the institutional animal care and use committee of Chungbuk National University (Permit Number: CBNUA-036-0902-01), and conducted the experiments in accordance with NIH guidelines for care and use of laboratory animals.

### Statistical analysis

**Figure 1:**
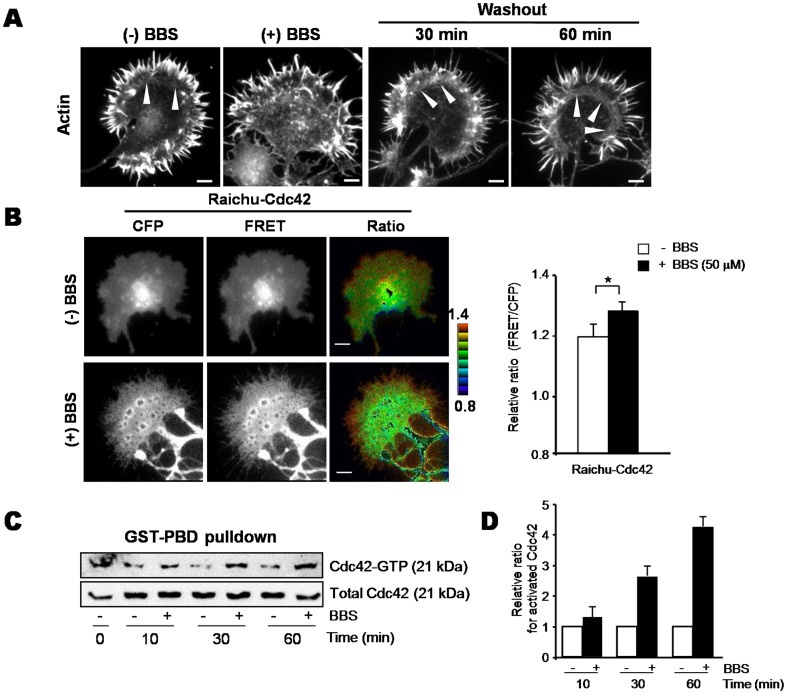
Blebbistatin alters growth cone actin dynamics through Cdc42 activation in cultured hippocampal neurons. A. Cultured hippocampal (HP) neurons at 2 days *in vitro* (DIV) were incubated with or without 50 µM blebbistatin (BBS) for 30 min, and then BBS was removed. Growth cones were stained for actin with TRITC-labeled phalloidin. Arrowheads indicate actin arcs. Scale bar, 10 µm. B. HP neurons were transfected with the Raichu-Cdc42 probe for 1 day and then incubated with or without 50 µM BBS for 10 min. Representative ratio images of FRET/CFP after BBS treatment are shown in the intensity-modulated display (IMD) mode (left). Bar graphs represent the relative emission ratio (FRET/CFP) of the whole cell area (right). The number of cells examined for each sample was 28 (with BBS) or 49 (without BBS). Error bars are ± SD. *, *P*<0.05. C. GST-PBD pulldown assay for activated Cdc42. HP neurons were incubated with or without 50 µM BBS for the indicated times and then lysed. Equal amounts of protein from each lysate were incubated with GST-PBD immobilized on glutathione-Sepharose. Total and GST-PBD–bound Cdc42 was probed by immunoblotting with anti-Cdc42 antibody. The data are representative of three independent experiments. D. Quantitative analysis of immunoblots from three independent experiments shown in C. The data were quantified by densitometric analysis using Image J software.

**Figure 2:**
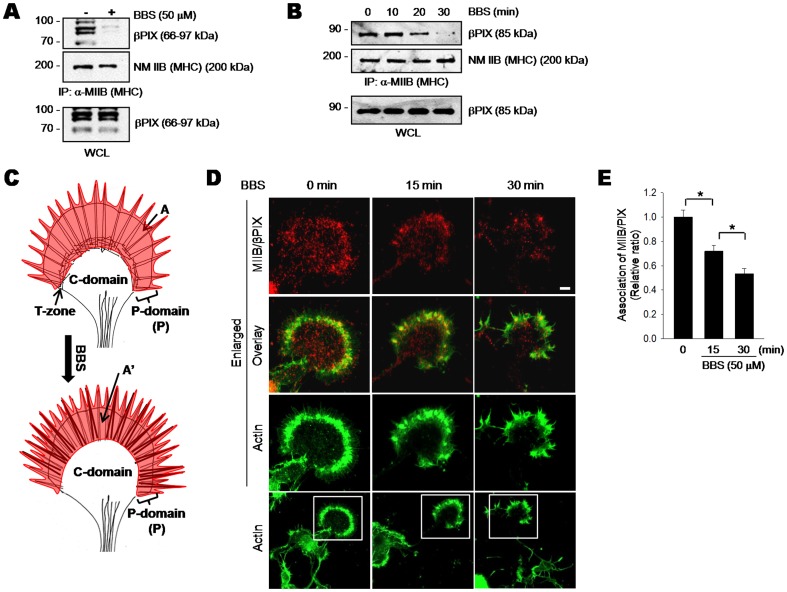
Blebbistatin dissociates βPIX and NM IIB in growth cones. A. BBS-induced dissociation of NM IIB–βPIX complex. Cultured HP neurons were incubated with or without 50 µM BBS for 30 min. Lysates were immunoprecipitated with anti-NM IIB antibody, and immunoblotted for βPIX and NM IIB. To ensure equal loading, lysates were immunoblotted with anti-βPIX antibody. B. Time-dependent dissociation of the NM IIB–βPIX complex. PC12 cells were incubated with 50 µM BBS for the indicated times. Lysates were processed as described above. C. Schematic diagram of the growth cone zones used for quantification of the NM IIB–βPIX interaction. Total actin–stained areas, A and A′, are shaded in red. D. *In situ* proximity ligation assay. HP neurons were processed using the Duolink *In Situ* Detection Reagents. Anti-βPIX and NM IIB antibodies were used as primary antibodies. Red spots represent the interaction of NM IIB and βPIX. To visualize actin structures in growth cones, cells were stained with Alexa Fluor 488–conjugated phalloidin (green). Scale bar, 10 µm. E. Quantification of association of βPIX with NM IIB. Fluorescence intensity of the NM IIB–βPIX complex before and after BBS treatment was expressed as a relative ratio of A′/A. The relative ratio in the BBS-untreated growth cones was set to 1. The number of cells examined was as follows: for time 0, *n* = 20; for 15 min, *n* = 25; for 30 min, *n* = 25. Error bars are ± SD. *, *P*<0.05.

**Figure 3:**
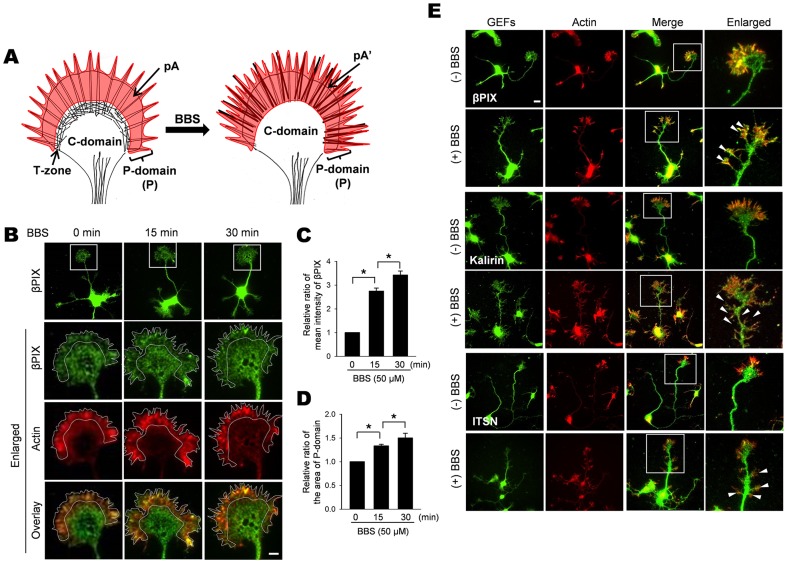
Blebbistatin alters localization of GEFs. A. Schematic diagram of growth cone zones used for quantification of GEF localization. Peripheral actin-stained areas, pA and pA', are shaded in red. B. Time course of βPIX localization in the actin-positive area after BBS treatment. Cultured HP neurons were incubated with 50 µM BBS for the indicated times and then double-stained with TRITC-labeled phalloidin for actin (red) and anti-βPIX antibody (green). The dotted lines indicate the peripheral actin–positive area. Scale bar, 10 µm. C. Quantification of βPIX localization in the peripheral actin–positive area. Mean fluorescence intensity of βPIX after BBS treatment was expressed as a relative ratio of pA'/pA. The mean intensity for pA in the BBS-untreated growth cones were set to 1. D. Quantification of the peripheral actin–positive area. The size of the peripheral actin–positive area after BBS treatment was expressed as a relative ratio of pA'/pA. The size for pA in the BBS-untreated growth cones were set to 1. The number of cells examined was as follows: for time 0, *n* = 12; for 15 min, *n* = 10; for 30 min, *n* = 10. Error bars are ± SD. *, *P*<0.05. E. BBS-induced alterations in localization of GEFs in a distal axon. Cultured HP neurons were incubated without or with 50 µM BBS for 30 min and co-stained for actin (red) and the indicated GEF (green). White arrowheads indicate the ends of branched neurites on the distal axon. Note the absence of branching on the distal axon in BBS-untreated cells. Scale bar, 10 µm.

**Figure 4:**
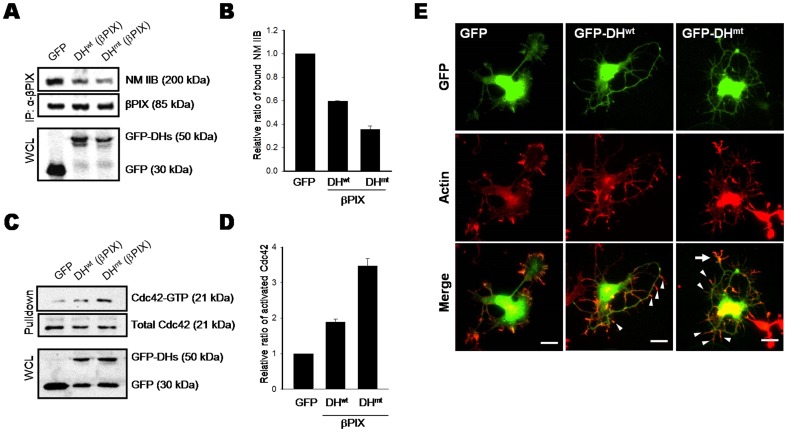
The effect of overexpression of the βPIX DH domain on growth cone formation and neurite branching. A. PC12 cells were transfected with plasmids encoding GFP (control), GFP-βPIX DH^wt^, or GFP-βPIX DH^mt^. Lysates were immunoprecipitated with an anti-βPIX monoclonal antibody, and immunoprecipitates were probed for NM IIB or βPIX (top). To monitor the expression of transfected genes, immunoblotting for GFP was performed (bottom). The blot is representative of three independent experiments. B. Quantitative analysis of immunoblots from three experiments shown in A. C. GST-PBD pulldown assay for Cdc42 activation. Total (input) and GST-PBD–bound Cdc42 was immunoblotted with an anti-Cdc42 antibody (top). The expression of transfected genes was monitored by immunoblotting for GFP (bottom). The blot is representative of three independent experiments. D. Quantitative analysis of immunoblots from three experiments shown in C. E. Cultured HP neurons were transfected with plasmids encoding GFP (control), GFP-DH^wt^ or GFP-DH^mt^. Cells were stained for actin (red), and expression of transfected GFP constructs (green) was examined by fluorescence microscopy. Scale bar, 10 µm.

We analyzed the data by the independent *t*-test (Figs. 1B and S1B) or Mann–Whitney *U*-test (Figs. 2C, 3C, 3E and 4C) using the SPSS software for Windows version 20.0 (IBM Corporation, Armonk, NY, USA); statistical significance was set at *p*<0.05. All values are expressed as mean±standard deviation (SD).

## Results

### Inhibition of NM II by BBS induced abnormal filopodial actin structures in growth cones

To understand the mechanism by which NM II regulates reorganization of the actin cytoskeleton in growth cones, we incubated cultured hippocampal cells with BBS (Fig. 1A). As shown in Fig. 1A, BBS-treated growth cones displayed filopodial structures that were more irregular in their length and thickness and more randomly oriented than those in untreated growth cones. Filopodial actin dynamics were more apparent in the movie (Movie S1). This effect of BBS was reversible, as evidenced by the reappearance of regular filopodial structures at the periphery and an actin arc in the T-zone after BBS removal, which critically depends on NM II activity. To examine whether these altered actin structures might be due to BBS-induced activation of Cdc42/Rac1, we performed FRET analysis using Raichu-Cdc42 and Rac1 probes (Fig. 1B, S1A and S1B) and a GST-PBD pulldown assay using the p21-binding domain (PBD) of p21-activated kinase 1 (PAK1) (Fig. 1C, 1D and S1C). In the absence of BBS, FRET for Cdc42 was weakly positive throughout the P-domain of the growth cone (Fig. 1B). BBS treatment induced a strong localized Cdc42 activation at the leading edge of the periphery. In line with FRET analysis, a GST-PBD pulldown assay revealed a marked increase in Cdc42 activity 30–60 min after BBS addition (Fig. 1C and 1D). We also performed FRET analysis and a GST-PBD pulldown assay to measure Rac1 activation, but did not detect any significant changes in FRET and GTP-bound Rac1 (Fig. S1). These results indicate that BBS treatment mainly stimulates Cdc42 activation, which results in dynamic alterations in filopodial structures.

### Inhibition of NM II dissociated GEFs and NM IIB in growth cones

Our previous study revealed an interaction between NM IIB and GEFs of the Dbl family [16]. Upon NM II inactivation by BBS, GEFs are released from NM II and their activity is enhanced. Therefore, we speculated that BBS-induced activation of Cdc42 in growth cones might occur by a similar mechanism. To test this idea, we checked whether BBS treatment dissociated NM IIB and GEFs in neuronal cells (Fig. 2A and 2B). βPIX is a major GEF in cultured hippocampal neurons and PC12 cells [21], [22]. Hippocampal cells express several βPIX isoforms, whereas PC12 cells mainly express the βPIXa isoform. First, we used co-immunoprecipitation to examine the effect of BBS on the interaction between NM IIB and βPIX. βPIX markedly dissociated from NM IIB in both cell types after BBS treatment (Fig. 2A and 2B). This βPIX release occurred in a time-dependent manner in PC12 cells (Fig. 2B). Next, to obtain spatial information on the βPIX release from NM IIB in growth cones, we examined the effect of BBS on colocalization of these proteins by using PLA, which links two different molecules that are in close proximity, and makes them detectable by fluorescence microscopy (Fig. 2D and 2E). βPIX and GIT strongly associate and exist as a heterodimer in cells [23]. As expected, strong fluorescence of the βPIX-GIT complex was detected as red dots throughout the growth cones regardless of BBS treatment (Fig. S2A), which demonstrated the feasibility of this assay to monitor protein-protein interactions in growth cones. Because NM IIB is located in the P-domain and the T-zone, we quantified changes in colocalization of NM IIB and βPIX in the actin-positive area (A and A' in Fig. 2C). No fluorescence was detected without primary βPIX antibody (Fig. S2B). Fluorescence indicating the interaction between NM IIB and βPIX was detectable in BBS-untreated growth cones, and was particularly noticeable along the T-zone (Fig. 2D). Fluorescence intensity in the actin-positive area gradually decreased and reached 51% of that in the untreated growth cones 30 min after BBS addition (Fig. 2E). Another GEF, kalirin, showed a similar response to BBS treatment, but its dissociation occurred to a lesser extent (by 17% percentage points) than in the case of βPIX (Fig. S3). These results clearly demonstrate a role of BBS-induced inactivation of NM II as a driving force in dissociating GEFs from NM IIB in growth cones.

### Inhibition of NM II induced redistribution of GEFs in growth cones and the distal axon

NM IIB is abundant in the T-zone and to a lesser extent in the P-domain (Fig. 3A). It is thus conceivable that BBS-induced dissociation of GEFs from NM IIB may cause a net flux of GEFs from the T-zone to the P- and C-domains. Free GEFs released in the P-domain, particularly in the vicinity of filopodia, may activate Cdc42, which would increase the size of the P-domain due to increase in actin polymerization. To test this idea, we quantified the effect of BBS on alterations in both the GEF levels and the size of the P-domain before and after BBS treatment (Fig. 3A). Because actin is a major cytoskeletal protein in the P-domain and could reflect its size, we replaced it by the peripheral actin–positive area (indicated by pA and pA' in Fig. 3A and by the dotted lines in Fig. 3B and S4A). BBS treatment increased localization of both βPIX and kalirin in the periphery in a time-dependent manner (Fig. 3C and S4B). The mean intensity of βPIX fluorescence increased 3.2-fold 30 min after BBS treatment compared to untreated controls (Fig. 3C), whereas the mean intensity of kalirin fluorescence increased 2.8-fold (Fig. S4B). BBS treatment also increased the size of the peripheral actin–positive area in a time-dependent manner (Fig. 3D and S4C), excluding the possibility that the increased localization of GEFs might result from a reduction in the area of P-domain. Together, the results support the idea that BBS dissociates GEFs from NM IIB and induces their relocation to peripheral actin. This might explain the BBS-induced Cdc42 activation and stimulation of filopodial dynamics.

To further investigate the effect of NM II inactivation on the distribution of GEFs in the distal axon, we treated cultured hippocampal neurons with BBS (Movie S2). Most untreated distal axons did not display any apparent projections. In contrast, BBS treatment frequently induced protrusions and spikes on the distal axons, which eventually evolved into branched neurites after longer treatment (Movie S2). BBS-treated growth cones sometimes fragmented into smaller ones, which later developed into neurite-like processes. This phenomenon might occur due to the ‘disto-proximal' migration of growth cone lamellipodia into the axon shaft [24]. Alternatively, the BBS-induced dissociation of GEFs from NM II, perhaps primarily the NM IIA isoform in the central domain, may result in aberrant targeting of GEFs to the distal axon. Thus, we performed immunocytochemistry to localize βPIX, kalirin and ITSN. These GEFs were detected in the protrusions and spikes on the distal axon (arrowheads in Fig. 3D). These results suggest that the BBS-induced release of GEFs from NM II results in their aberrant targeting to the distal axon. Subsequent activation of Cdc42 might trigger growth of small protrusions, which develop into mature neurites with the participation of microtubules.

### Disruption of the NM II–GEF interaction affects growth cone dynamics

To determine whether NM II regulates actin dynamics in the growth cone and distal axon by interacting with the Dbl family GEFs, we introduced the DH domain of βPIX, which is an NM II–binding region, into cells and analyzed its effect (Fig. 4). Because the DH domain has catalytic GEF activity and its overexpression might affect other cellular functions independently of the NM II–βPIX interaction, we also used a catalytically inactive version of the βPIX DH domain as a control. As illustrated in Fig. 4A and 4B, both the wild-type (DH^wt^) and catalytically inactive mutant (DH^mt^, L238R/L239R) forms significantly blocked the interaction between NM IIB and endogenous βPIX in PC12 cells. Moreover, overexpression of the βPIX DH^wt^ or DH^mt^ domain stimulated Cdc42 activation, as determined by a GST-PBD pulldown assay (Fig. 4C and 4D). Next, we examined the morphological changes in hippocampal neurons induced by overexpression of the βPIX DH domain (Fig. 4E). The most striking feature was the development of multiple protrusions on both axonal and dendritic shafts. The axons were unable to extend in a specific direction due to multiple bends. Consequently, the axons circled around the cell body. Not surprisingly, growth cone structures were seldom observed at these axon terminals, suggesting that the NM II–GEF interaction also controls growth cone formation. Neurite thickness was also significantly reduced in DH-expressing cells. In some instances, the appearance of multiple small neurites made it difficult to morphologically distinguish axons from dendrites. Control cells (expressing GFP only) showed linear extension of thick axons with growth cones at their ends (Fig. 4E, left). To confirm that this phenomenon was not specific to the βPIX DH domain, we performed a similar experiment with a DH domain derived from Tiam1, another Dbl family GEF. Overexpression of the wild-type or catalytically inactive mutant form of the Tiam1 DH domain induced dissociation of the NM IIB–Tiam1 complex and similar morphological changes (Fig. S5). These results suggest that overexpression of DH domains interferes with the NM II–GEF complexes and induces aberrant targeting of activated GEFs, which leads to generation of multiple protrusions and branches on both axons and dendrites.

**Figure 5:**
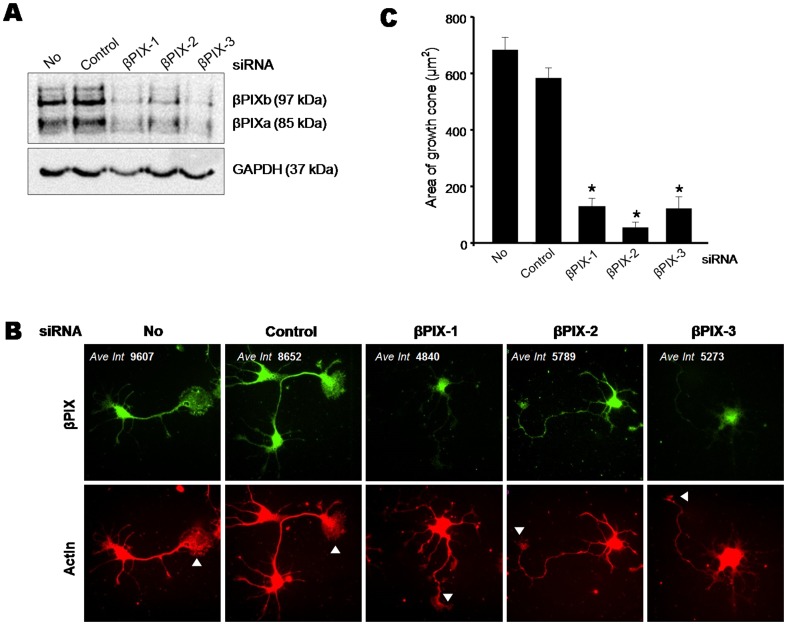
βPIX is required for growth cone and neurite formation in HP neurons. A. Cultured HP neurons at DIV 2 were transfected with control siRNA or three βPIX-specific siRNAs for 3 days. Lysates were subjected to immunoblotting for βPIX (top) and GAPDH (bottom). B. Cells were transfected as described above and co-stained for βPIX (green) and actin (red). Note small growth cone–like structures at the neurite tips of βPIX siRNA–treated cells (white arrowheads). C. The area of the growth cones in untreated, control siRNA- and βPIX siRNA–treated cells was measured using the MetaMorph software. Error bars are ± SD. *, *P*<0.05.

As the above results suggested the importance of βPIX as a partner of NM II in growth cones, we investigated the effect of βPIX depletion on growth cone formation. For this purpose, we used three different siRNAs to obtain specific βPIX depletion (Fig. 5A). These siRNAs almost completely knocked down two major βPIX isoforms, the short isoform βPIXa and the longer isoform βPIXb. Fluorescence intensity of βPIX in βPIX siRNA–treated cells was also markedly decreased, as indicated by immunocytochemical analysis (Fig. 5B, top). Growth cone formation in these cells was severely compromised; only the remnants of growth cone–like structures could be detected (compare arrowheads in Fig. 5B, bottom). Consistent with this observation, quantification of the area of growth cone–like structures revealed its marked decrease in βPIX siRNA–treated cells (Fig. 5C). Moreover, βPIX siRNA–treated hippocampal neurons showed longer and thinner axons than control siRNA–treated neurons.

### Activation of Cdc42 or Rac1 induces NM II disassembly and leads to dissociation of the NM II–GEF complex

**Figure 6:**
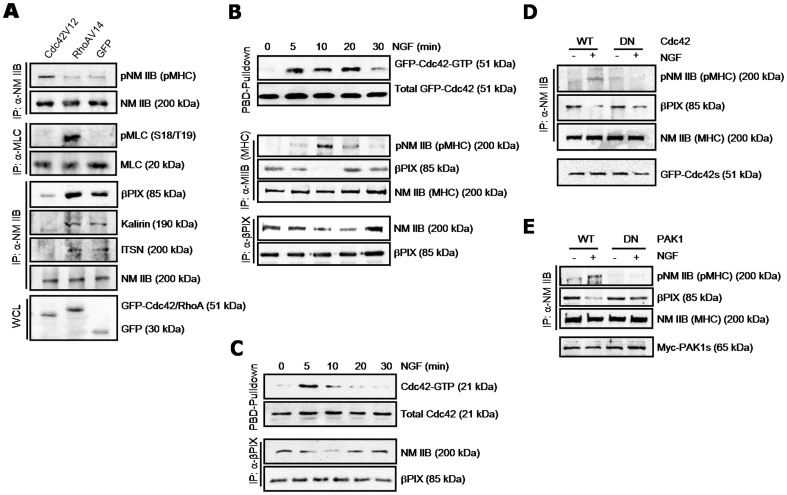
Cdc42–PAK1 pathway mediates dissociation of the NMII-GEF complex in the NGF signaling pathway. A. PC12 cells were transfected with GFP-tagged constructs for active Cdc42 (Cdc42V12), RhoA (RhoAV14) or GFP alone for 24 h. Lysates were immunoprecipitated with anti-NM IIB or anti-MLC antibodies, and immunoprecipitates were immunoblotted for phosphorylated and total NM IIB (top), phosphorylated (Ser18/Thr19) and total MLC (2nd panel), or the indicated GEFs (3rd panel). The expression of transfected genes was assessed by immunoblotting for GFP (bottom). B. Cells were transfected with GFP-tagged wild-type Cdc42 and stimulated with 100 ng/ml NGF for the indicated times. A GST-PBD pulldown assay was performed to measure Cdc42 activation (top). NGF-stimulated lysates were immunoprecipitated with anti-NM IIB (middle) or βPIX (bottom) antibodies, and immunoprecipitates were immunoblotted for the indicated proteins. Phosphorylated NM IIB (pNM IIB) was detected by anti-phospho-threonine antibody. C. Non-transfected cells were stimulated with 100 ng/ml NGF for the indicated times. A GST-PBD pulldown assay was performed to measure Cdc42 activation (top). Lysates were immunoprecipitated with anti-βPIX antibody, and immunoprecipitates were immunoblotted for the indicated proteins (bottom). D and E. Cells were transfected with GFP-tagged constructs for WT or DN-Cdc42 (Cdc42N17) (D) or Myc-tagged constructs for WT or DN-PAK1 (H83/86L, K299R) (E), and then treated with or without 100 ng/ml NGF for 10 min. Lysates were immunoprecipitated with anti-NM IIB antibody, and immunoprecipitates were immunoblotted for the indicated proteins (top). The expression of transfected Cdc42 and PAK1 constructs was confirmed by immunoblotting with anti-GFP and anti-Myc antibody, respectively (bottom). The data are representative of three independent experiments.

Inhibition of NM II ATPase activity with BBS resulted in Cdc42 activation (Fig. 1). Activated Cdc42 may form a positive feedback loop, promoting dissociation of GEFs from NM II. We addressed this issue by examining whether the constitutively active form of Cdc42 (Cdc42V12) would dissociate the NM II–GEF complex in PC12 cells (Fig. 6A). In the presence of Cdc42V12, βPIX, kalirin and ITSN were almost completely dissociated from NM IIB (Fig. 6A, 3rd panel). In contrast, no significant dissociation was detected in cells expressing the constitutively active form of RhoA (RhoAV14) or GFP alone. The assembly and disassembly of the actin–myosin II complex is regulated by two factors: phosphorylation of the regulatory light chain of myosin (MLC) and of MHC [25], [26]. MLC phosphorylation causes an increase in the ATPase activity of myosin, which promotes actin–myosin II assembly. In contrast, MHC phosphorylation induces MHC filament disassembly accompanied by local disassembly of actin–myosin II complexes [27]–[32]. We therefore tested whether Cdc42V12 might influence phosphorylation of NM IIB MHC or MLC. Cdc42V12 induced MHC phosphorylation but not MLC phosphorylation (Fig. 6A, 1st and 2nd panels). No significant MHC phosphorylation was observed in cells expressing RhoAV14 or GFP. RhoAV14 induced a marked phosphorylation of MLC, as previously reported [33]. These results suggest that constitutive activation of Cdc42 may form a positive feed-forward loop by amplifying the release of GEFs from NM II via MHC disassembly, which would result in further Cdc42 activation.

Next, we determined whether activation of Cdc42 or Rac1 plays a similar role in a physiological context. Nerve growth factor (NGF) is the founding member of neurotrophins, and can stimulate activation of both Cdc42 and Rac1 [34]. Thus, we determined whether NGF facilitates GEF dissociation from NM II in a manner dependent on the activation of these GTPases (Fig. 6B and S6). Signals were amplified by overexpression of GFP-tagged wild-type Cdc42 or Rac1. In Cdc42-overexpressing cells NGF-stimulated Cdc42 activation was apparent at 5 min and was sustained up to 20 min after NGF addition (Fig. 6B, top). At 30 min, Cdc42 activation decreased to nearly the basal level. The phosphorylation levels of MHC in NM IIB peaked at 10 min and returned to the prestimulation level at 30 min (Fig. 6B, middle). Dissociation of the NM IIB–βPIX complex was maximal at 10–20 min, and paralleled MHC phosphorylation (Fig. 6B bottom). Similarly, the NM IIB–βPIX complex dissociated in Rac1-overexpressing cells in response to NGF stimulation, but with slightly different kinetics (Fig. S6). Rac1 activation began at 5 min and continued until 30 min (Fig. S6, top). Although MHC phosphorylation was first detected at 20 min, it also paralleled the dissociation of the NM IIB–βPIX complex (Fig. S6, bottom). To test whether NGF-induced dissociation of the NM IIB–βPIX complex correlates with Cdc42 activation in non-transfected cells, we performed a GST-PBD pulldown and immunoprecipitation analysis (Fig. 6C). In contrast to sustained activation of Cdc42 in Cdc42-transfected cells, Cdc42 activation was transient in non-transfected cells, and was observed 5 min after NGF addition (Fig. 6C, top), whereas dissociation of the NM IIB–βPIX complex occurred 10 min after NGF addition stimulation (Fig. 6C, bottom). Both Cdc42 activation and dissociation of the NM IIB–βPIX complex showed similar kinetics in wild-type Cdc42–transfected and non-transfected cells. We next investigated whether the dominant negative forms of Cdc42 (DN-Cdc42) or p21-activated kinase1 (DN-PAK1), which is a downstream effector of Cdc42, would block the NGF-stimulated phosphorylation of MHC and dissociation of the NM II–βPIX complex (Fig. 6D). MHC phosphorylation and dissociation of the NM II–βPIX complex occurred in response to NGF stimulation in cells expressing wild-type Cdc42 (WT-Cdc42) but not DN-Cdc42. Similarly, expression of DN-PAK1 almost completely suppressed MHC phosphorylation and dissociation of the NM II–βPIX complex (Fig. 6E). Taken together, these results support the notion that the NGF-induced dissociation of GEFs from NM II occurs downstream of Cdc42 or Rac1 activation–MHC phosphorylation.

## Discussion

**Figure 7:**
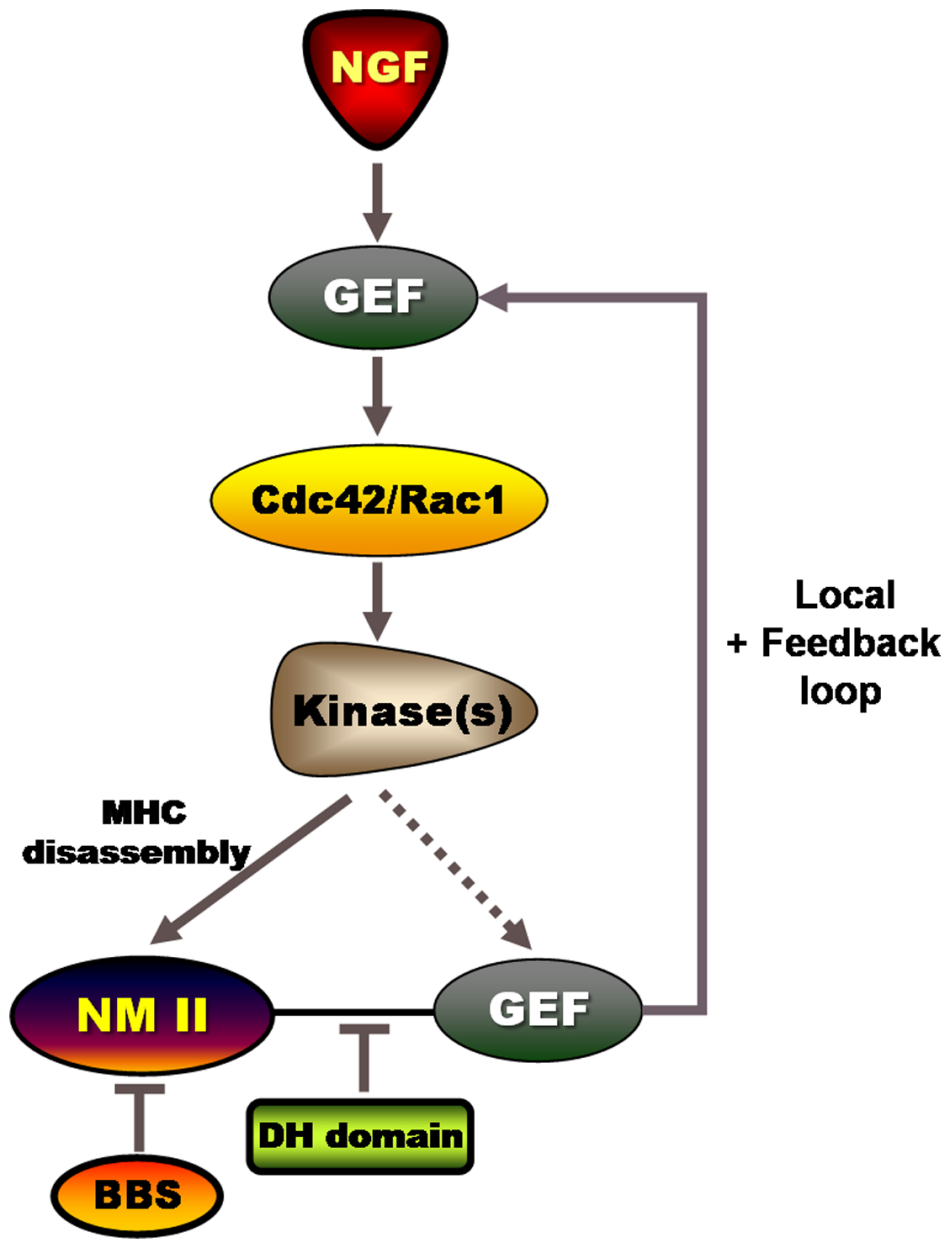
Model for regulation of growth cone morphology and neurite branching by NM II through dynamic interactions with GEFs. NM II forms a complex with GEFs of the Dbl family when it is active as an ATPase [16]. The scheme shows three different ways to disrupt this complex: two non-physiological ones (inhibition of NM II using BBS or DH domain overexpression) and a physiological one (stimulation with NGF). Non-physiological disruptions directly target NM II and its binding interface with GEFs, whereas physiological disruption by NGF stimulation operates through the GEF–Cdc42 (Rac1)–PAK1 pathway. BBS or DH domain overexpression induces persistent dissociation of the NM II–GEF complex, which results in strong Cdc42 activation. Activation of this Rho GTPase in turn promotes the release of GEFs from NM II, thereby forming a positive feedback loop. The resultant aberrant targeting of GEFs, concomitant with Rho GTPase activation, alters actin dynamics, which leads to morphological abnormalities in growth cones and on the distal axon, such as irregular filopodial structures, and even fragmentation of growth cones and multiple protrusions and branching on the distal axon. In contrast to these non-physiological disruptions, NGF stimulates transient Cdc42/Rac1 activation; thus, the dissociation of the NM II–GEF (for example βPIX) complex is also transient. We believe that this transient and regulated mode of Rho GTPase activation meets the requirement for physiological growth cone motility and neurite outgrowth associated with neuronal differentiation.

In this study, we demonstrated that NM II is able to modulate neuronal morphology through GEFs of the Dbl family in three different ways: when NM II ATPase activity is pharmacologically inhibited by BBS, when the association between NM II and GEFs is inhibited by overexpression of the DH domain (which is a binding interface for NM II), and upon NGF stimulation (Fig. 7). All these treatment modalities resulted in dissociation of the NM II–GEF complexes by a direct action or indirectly through activation of Cdc42 and/or Rac1. Long-term BBS treatment and overexpression of the DH domain induced a global effect because of persistent aberrant targeting of GEFs. Because of dysregulated actin dynamics, growth cones and distal axons often showed abnormal morphological changes: no formation of growth cones or their fragmentation, and multiple protrusions and branches on the shafts of distal axons. This NM II assembly/disassembly–dependent mechanism of Cdc42/Rac1 activation is analogous to regulation of the activity of RhoA-specific GEFs–RhoA in a microtubule assembly/disassembly–dependent manner [35], [36]. In contrast, NGF transiently disassembled NM II via the Cdc42/Rac1–PAK pathway and released GEFs locally, thereby inducing a physiological response that included neuronal differentiation.

Precise spatio-temporal regulation of Rho GTPases is crucial for determining the morphology of growth cones and the distal axon. The contractility of NM II is another key factor in maintaining their proper morphology, as demonstrated by the BBS-induced disruption of the actin arc in the transition zone of growth cones. However, our data suggest an additional regulatory mechanism(s) for NM II in this respect. BBS treatment slows retrograde actin flow in growth cones; 50% of this effect is caused by the loss of NM II contractility and ∼30% by pushing by actin polymerization at the leading edge [13]. There has been no mechanistic explanation for BBS-induced activation of Cdc42 and the resultant increase in filopodial actin structures. Because Cdc42 is activated downstream of Dbl family GEFs, which colocalize with NM II in growth cones (Fig. 2), there must be a link between BBS-induced inhibition of NM II and activation of Dbl family GEFs. Our previous study suggested a potential mechanism of the regulation of the catalytic activity of Dbl family GEFs by NM II, which relies on NM II–GEF binding [16]. Previous studies demonstrated that Cdc42 and Rac1 are activated in a manner distinct from the above mechanism in the growth cones of NGF-stimulated PC12 cells and N1E-115 neuroblastoma cells [34], [37]. In line with these observations, our FRET analysis showed activation of both Cdc42 and Rac1 in growth cones of cultured hippocampal neurons even without BBS treatment. However, when NM II was inhibited by BBS, only Cdc42 was specifically activated, and its activation was restricted to the peripheral edge of growth cones. In contrast to filopodial extensions in growth cones of *Aplysia* [12] and cultured embryonic chicken neurons [38], we observed rather short and thick filopodia that did not display a uniform centrifugal direction. Adding to the complexity, RhoA might be involved in BBS-induced filopodial dynamics, as evidenced by its localized activity in the peripheral domain of growth cones [37], although we did not pursue RhoA activation. BBS-treated fibroblasts show a substantial increase in lamellipodial area, which suggests Rac1 activation [36]. In line with this observation, Rac1 was significantly activated in NM IIA knockout embryonic fibroblasts [36]. These collective results of different studies on Cdc42- or Rac1-specific activation in response to BBS treatment might reflect species- or cell type–specific differences. The BBS-stimulated expansion of microtubules might partially explain the specificity of BBS-induced Rho GTPase activation [38], [39]. The interaction of a GEF(s) with microtubules and its subsequent translocation to the correct area might determine which Rho GTPase would be activated in that area. Further studies are warranted to clarify the underlying mechanism of the BBS-stimulated activation of specific Rho GTPases.

The essential catalytic unit of Dbl family GEFs consists of a DH domain and an adjacent pleckstrin-homology (PH) domain. The latter recruits Rho GTPases for efficient exchange of GDP for GTP [40], [41]. The isolated DH domain thus shows lower catalytic activity than the tandem DH–PH module [42]. In cells, the GEF activity of the DH domain in the full-length protein is stimulated by diverse ways including phosphorylation and protein-protein interactions in response to external stimuli. We therefore assume that even the wild-type βPIX DH domain has no substantial GEF activity toward Cdc42 and Rac1. Then, how can the βPIX DH domain alone (either wild-type or the dominant negative mutant) cause Cdc42 activation concomitant with morphological changes? Our interpretation is that forced expression of the DH domain might indirectly activate endogenous βPIX or other GEFs by disrupting the NM II–βPIX interaction, as shown in Fig. 2. Indeed, the DH domain, conserved in all Dbl family GEFs including βPIX, is the binding site for NM II [16]. The βPIX DH–PH module may transiently interact with Rho GTPases, but a stable interaction is mediated by the GIT-binding domain [43] and/or SH3 domain [44]. Thus, our current understanding is that the βPIX DH domain provides a site for relatively stable binding of NMII but not Rho GTPases. However, we cannot completely exclude other possible explanations of how the βPIX DH domain may activate Cdc42 regardless of its lack of GEF activity. Concerns regarding the specificity of the βPIX DH domain still remain. To address this issue, we used a DH domain from a different protein, Tiam1. Hippocampal neuron cells expressing DH^wt^ or DH^mt^, regardless of their sources, displayed common morphological features: no apparent growth cones and multiple slender neurites with filopodial protrusions and branches on the shafts. These results, combined with the co-immunoprecipitation data, strongly suggest that the DH domain functions predictably as a disintegrator of the NM II–GEF complex in cells. In axon branching, the initial filopodial extension is normally accompanied by the invasion of microtubules and, later, by further extension of collateral branches. Most of the DH domain–expressing cells, however, exhibited small transient filopodial sprouting and branches without apparent growth cones at their tips, suggesting dysregulation of Rho GTPase activation. βPIX depletion consistently induced similar features: no apparent growth cones and long slender neurites (Fig. 5). This resembles a pathological condition in which excessive branching occurs on the shaft of mechanoreceptor axons following spinal cord injury [45]. Because balanced GEF activity, such as that of Vav2, is necessary for optimal neurite outgrowth and branching [46], forced expression of the DH domain may target GEFs aberrantly by disrupting the NM II–GEF complexes and thereby hampering proper branching and neurite outgrowth.

Although BBS treatment and expression of the DH domain are non-physiological approaches, they have some advantages because of their high potency. However, their drawback is that they directly target NM II. Therefore, using NGF as a stimulant, we explored the signaling pathway leading to dissociation of the NM II–GEF complex in PC12 cells. In the NGF signaling pathway, the dissociation of the NM II–βPIX complex occurred downstream of Cdc42 and Rac1 activation. This dissociation became more obvious in the presence of a constitutively active form of Cdc42: βPIX, kalirin and ITSN almost completely dissociated from NM II (Fig. 6). Conversely, a dominant negative form of Cdc42 blocked NGF-stimulated MHC phosphorylation and NM II–βPIX dissociation. Interestingly, Cdc42/Rac1 activation was followed, with a 5–10 min lag, by MHC phosphorylation and dissociation of the NM II–βPIX complex. MHC phosphorylation induces the disassembly of NM II by inhibiting its motor activity and association with F-actin [26], [28], [29]. This would explain the 5–10 min lag between Cdc42/Rac1 activation and dissociation of the NMII–βPIX complex. On the basis of the above data, we suggest that Cdc42/Rac1 activation–dependent MHC phosphorylation induces NM II disassembly, and thus causes dissociation of the NM II–βPIX complex. PAK, a well-known effector of Cdc42 and Rac1, might mediate the function of these Rho GTPases by phosphorylating MHC. In line with this possibility, PAK inhibition by its dominant negative form suppressed NGF-stimulated MHC phosphorylation. BBS and the DH domains also induced Cdc42 activation, which resulted in active filopodial dynamics in growth cones and in multiple filopodia sprouting on the neurite shaft. Therefore, it is likely that PAK also mediates the positive effect of BBS and the DH domains on actin dynamics. Santiago-Medina and coworkers reported distinct expression of group I PAK isoforms, PAK1–3, in mammalian growth cones [47]. Interestingly, of these PAK isoforms, PAK1 localized at the filopodial tips of growth cones, suggesting its specific function in filopodial actin dynamics. Additionally, because group I PAKs form complexes with βPIX, and disruption of PAK-βPIX interaction affects growth cone motility and morphology [47], these PAKs may contribute to the positive feedback loop for the GEF–Cdc42/Rac1–kinase pathway (Fig. 7).

In conclusion, the present study provided evidence for a link between NM II and Cdc42/Rac1, whose activation generates a positive feedback loop in the regulation of neuronal morphology. This NM II-dependent regulatory mechanism may operate in neurite branching and angiogenic sprouting [48], where precise regulation of contractility and actin dynamics is critically important. Moreover, the depletion of cellular ATP induces actomyosin disassembly [49], which would result in an uncontrolled release of GEFs from NM II, accompanied by Rho GTPase activation. Hence, the NM II-dependent release of GEFs may contribute to the pathogenesis of cerebral ischemia by the Rac1-dependent generation of reactive oxygen species [50] and postischemic morphological changes [51].

## Supporting Information

Figure S1 **BBS induces no significant Rac1 activation in cultured HP neurons.** A. FRET analysis was performed to measure Rac1 activation using the Raichu-Rac1 probe in HP neurons. Cells were transfected with the Raichu-Rac1 probe for 24 h and then incubated with or without 50 µM BBS for 10 min. Representative ratio images of FRET/CFP after BBS treatment are shown in the intensity-modulated display (IMD) mode (top). B. Bar graphs represent the relative emission ratio (FRET/CFP) of the whole cell area (bottom). The number of cells examined for each sample was 30 (with BBS) or 40 (without BBS). C. GST-PBD pulldown assay for Rac1. HP neurons were incubated with or without 50 µM BBS for the indicated times and lysed. Equal amounts of protein from each lysate were incubated with GST-PBD immobilized on glutathione-Sepharose. Total and GST-PBD–bound Rac1 was probed by immunoblotting with anti-Rac1 antibody.
(TIF)

Figure S2 **Control experiments for the** ***in situ*** **proximity ligation assay.** A. HP neurons were incubated with BBS for the indicated times. Cells were immunostained for βPIX and GIT, a specific binding partner for βPIX. Cells were stained with Alexa Fluor 488–conjugated phalloidin for actin (green). B. HP neurons were incubated only with anti-NM IIB antibody to confirm the specificity of this ligation assay.
(TIF)

Figure S3 **Blebbistatin dissociates kalirin and NM IIB in growth cones.** A. *In situ* proximity ligation assay. HP neurons were processed using the Duolink *In Situ* Detection Reagents. Anti-kalirin and NM IIB antibodies were used as primary antibodies. Red spots represent the interaction of NM IIB and kalirin. To visualize actin structures in growth cones, cells were stained with Alexa Fluor 488–conjugated phalloidin (green). Scale bar, 10 µm. B. Quantification of association of kalirin with NM IIB. Fluorescence intensity of the NM IIB–kalirin complex before and after BBS treatment was expressed as a relative ratio of A′/A. The relative ratio in the BBS-untreated growth cones was set to 1. The number of cells examined was as follows: for time 0, *n* = 16; for 15 min, *n* = 18; for 30 min, *n* = 18. Error bars are ± SD. *, *P*<0.05.
(TIF)

Figure S4 **Blebbistatin alters localization of kalirin.** A. HP neurons were incubated with 50 µM BBS for the indicated times and then double-stained with TRITC-labeled phalloidin for actin (red) and anti-kalirin antibody (green). The dotted lines indicate the peripheral actin–positive area. Scale bar, 10 µm. B. Quantification of kalirin localization in the peripheral actin–positive area. Mean fluorescence intensity of kalirin after BBS treatment was expressed as a relative ratio of pA'/pA. The mean intensity for pA in the BBS-untreated growth cones was set to 1. C. Quantification of the peripheral actin–positive area. The size of the peripheral actin–positive area after BBS treatment was expressed as a relative ratio of pA'/pA. The size for pA in the BBS-untreated growth cones were set to 1. The number of cells examined was 22 (for time 0) or 25 (for 30 min). Error bars are ± SD. *, *P*<0.05.
(TIF)

Figure S5 **The effect of overexpression of the Tiam1 DH domain on growth cone formation and neurite branching.** A. PC12 cells were transfected with plasmids encoding GFP (control), GFP-Tiam1 DH^wt^, or GFP-Tiam1 DH^mt^ domain. Lysates were immunoprecipitated with anti-Tiam1 antibody, and immunoprecipitates were probed for NM IIB or Tiam1 (top). To assess the expression of transfected genes, immunoblotting for GFP was performed (bottom). The data are representative of three independent experiments. B. Quantitative analysis of immunoblots from three independent experiments shown in A. C. Cultured HP neurons were transfected with plasmids encoding GFP (control), GFP-DH^wt^ or GFP-DH^mt^ domain. Cells were stained for actin (red), and expression of transfected GFP constructs (green) was examined by fluorescence microscopy. Scale bar, 10 µm.
(TIF)

Figure S6 **NGF stimulates dissociation of the NMII–GEF complex through Rac1 activation.** A. PC12 cells were transfected with GFP-tagged Rac1 and stimulated with 100 ng/ml NGF for the indicated times. A GST-PBD pulldown assay was performed to measure Rac1 activation. B. NGF-stimulated lysates were immunoprecipitated with anti-NM IIB antibody, and immunoprecipitates were immunoblotted with anti-phospho-threonine antibody for pNM IIB, total NM IIB or βPIX antibodies. The data are representative of three independent experiments.
(TIF)

Movie S1 **BBS treatment enhances growth cone actin dynamics.** Cultured hippocampal neurons were incubated with 50 µM BBS and monitored for 1 h under Olympus IX81 inverted microscope with a CCD camera (Cascade 512B, Photometrics). Arrowheads indicate filopodial protrusions showing dynamic movements.
(WMV)

Movie S2 **BBS treatment induces multiple protrusions and branching on the distal axon.** Cultured hippocampal neurons were incubated with 50 µM BBS and monitored for 1 h under Olympus IX81 inverted microscope with a CCD camera (Cascade 512B). Arrowheads indicate distal axon protrusions and branching.
(WMV)
