# Supplementary figures and images for: Non-Muscle Myosin II Regulates Neuronal Actin Dynamics by Interacting with Guanine Nucleotide Exchange Factors
Source: PLoS One. 2014 Apr 21;9(4):e95212. doi: 10.1371/journal.pone.0095212 (PMC3994028; doi:10.1371/journal.pone.0095212)

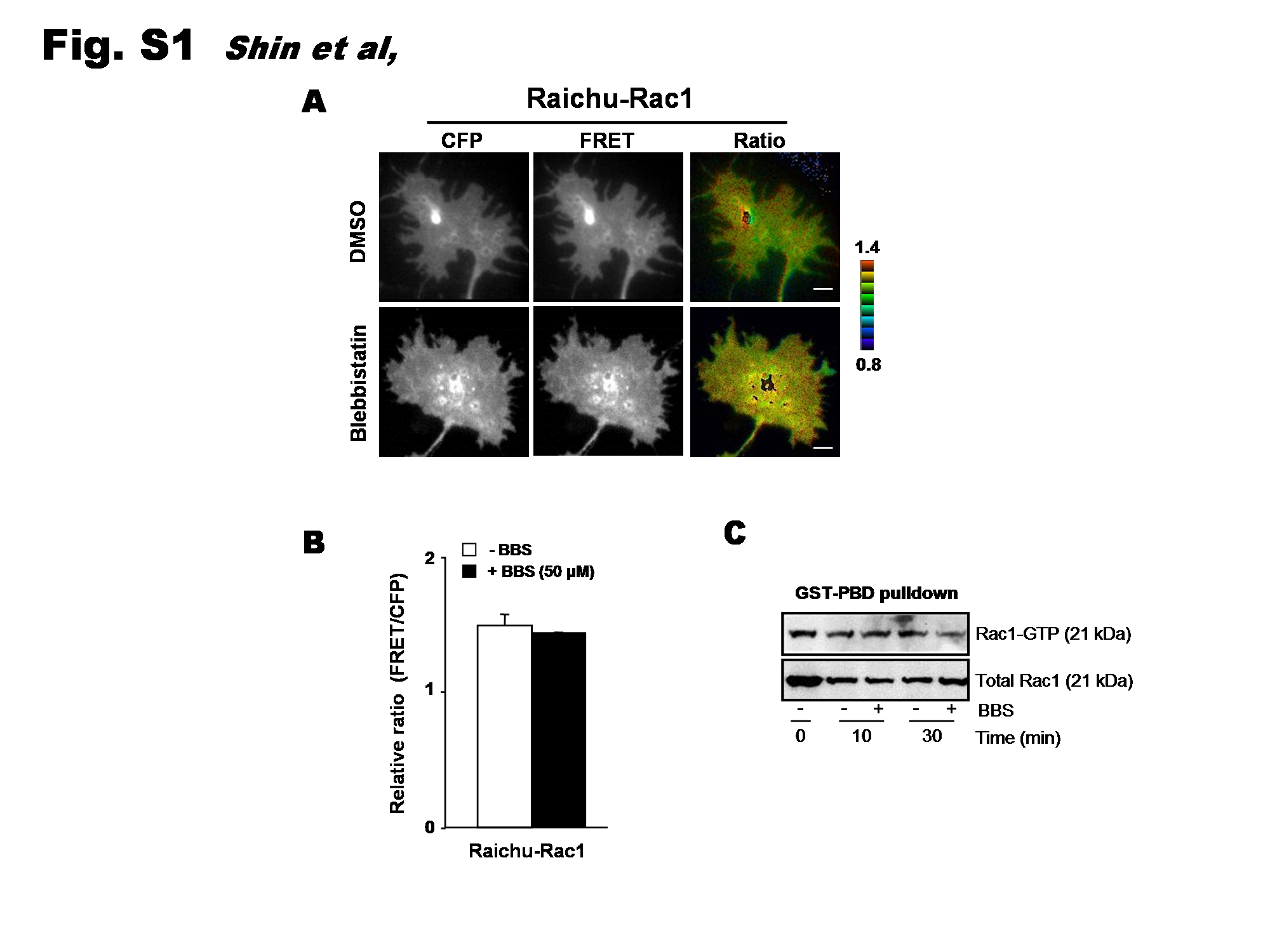

Supplement: Figure S1 — BBS induces no significant Rac1 activation in cultured HP neurons. A. FRET analysis was performed to measure Rac1 activation using the Raichu-Rac1 probe in HP neurons. Cells were transfected with the Raichu-Rac1 probe for 24 h and then incubated with or without 50 µM BBS for 10 min. Representative ratio images of FRET/CFP after BBS treatment are shown in the intensity-modulated display (IMD) mode (top). B. Bar graphs represent the relative emission ratio (FRET/CFP) of the whole cell area (bottom). The number of cells examined for each sample was 30 (with BBS) or 40 (without BBS). C. GST-PBD pulldown assay for Rac1. HP neurons were incubated with or without 50 µM BBS for the indicated times and lysed. Equal amounts of protein from each lysate were incubated with GST-PBD immobilized on glutathione-Sepharose. Total and GST-PBD–bound Rac1 was probed by immunoblotting with anti-Rac1 antibody. (TIF) [file pone.0095212.s001.tif]

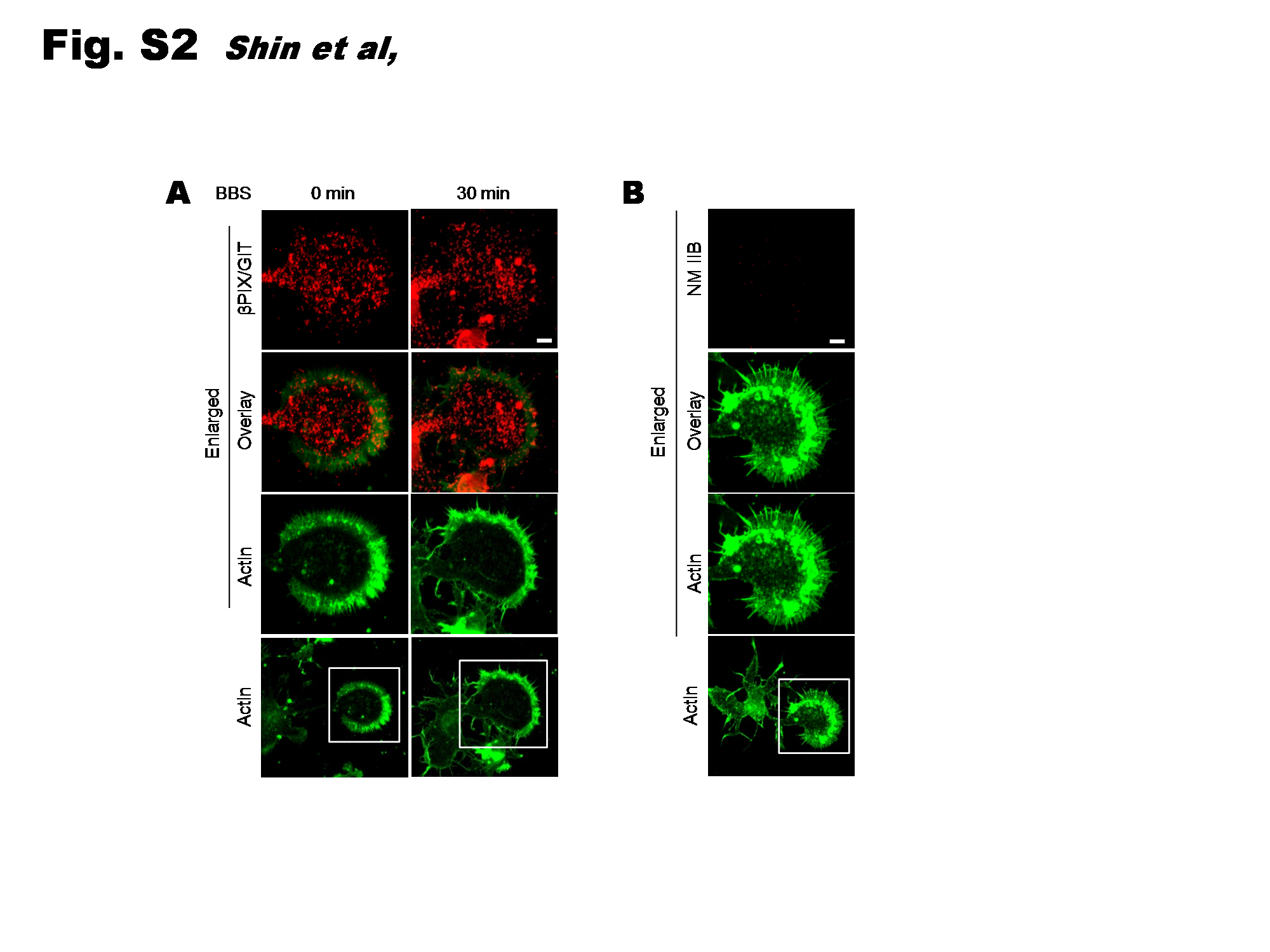

Supplement: Figure S2 — Control experiments for the in situ proximity ligation assay. A. HP neurons were incubated with BBS for the indicated times. Cells were immunostained for βPIX and GIT, a specific binding partner for βPIX. Cells were stained with Alexa Fluor 488–conjugated phalloidin for actin (green). B. HP neurons were incubated only with anti-NM IIB antibody to confirm the specificity of this ligation assay. (TIF) [file pone.0095212.s002.tif]

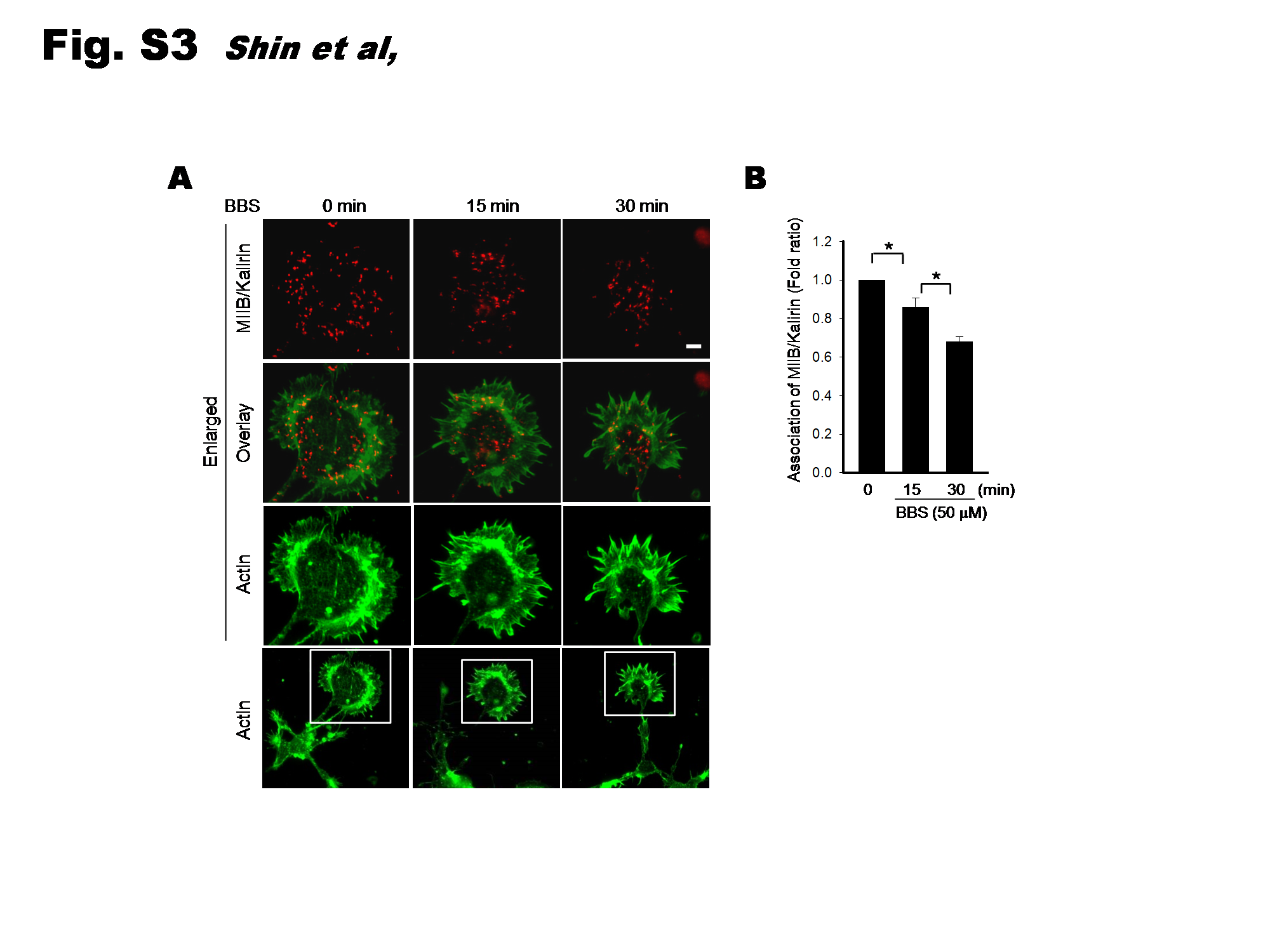

Supplement: Figure S3 — Blebbistatin dissociates kalirin and NM IIB in growth cones. A. In situ proximity ligation assay. HP neurons were processed using the Duolink In Situ Detection Reagents. Anti-kalirin and NM IIB antibodies were used as primary antibodies. Red spots represent the interaction of NM IIB and kalirin. To visualize actin structures in growth cones, cells were stained with Alexa Fluor 488–conjugated phalloidin (green). Scale bar, 10 µm. B. Quantification of association of kalirin with NM IIB. Fluorescence intensity of the NM IIB–kalirin complex before and after BBS treatment was expressed as a relative ratio of A′/A. The relative ratio in the BBS-untreated growth cones was set to 1. The number of cells examined was as follows: for time 0, n = 16; for 15 min, n = 18; for 30 min, n = 18. Error bars are ± SD. *, P<0.05. (TIF) [file pone.0095212.s003.tif]

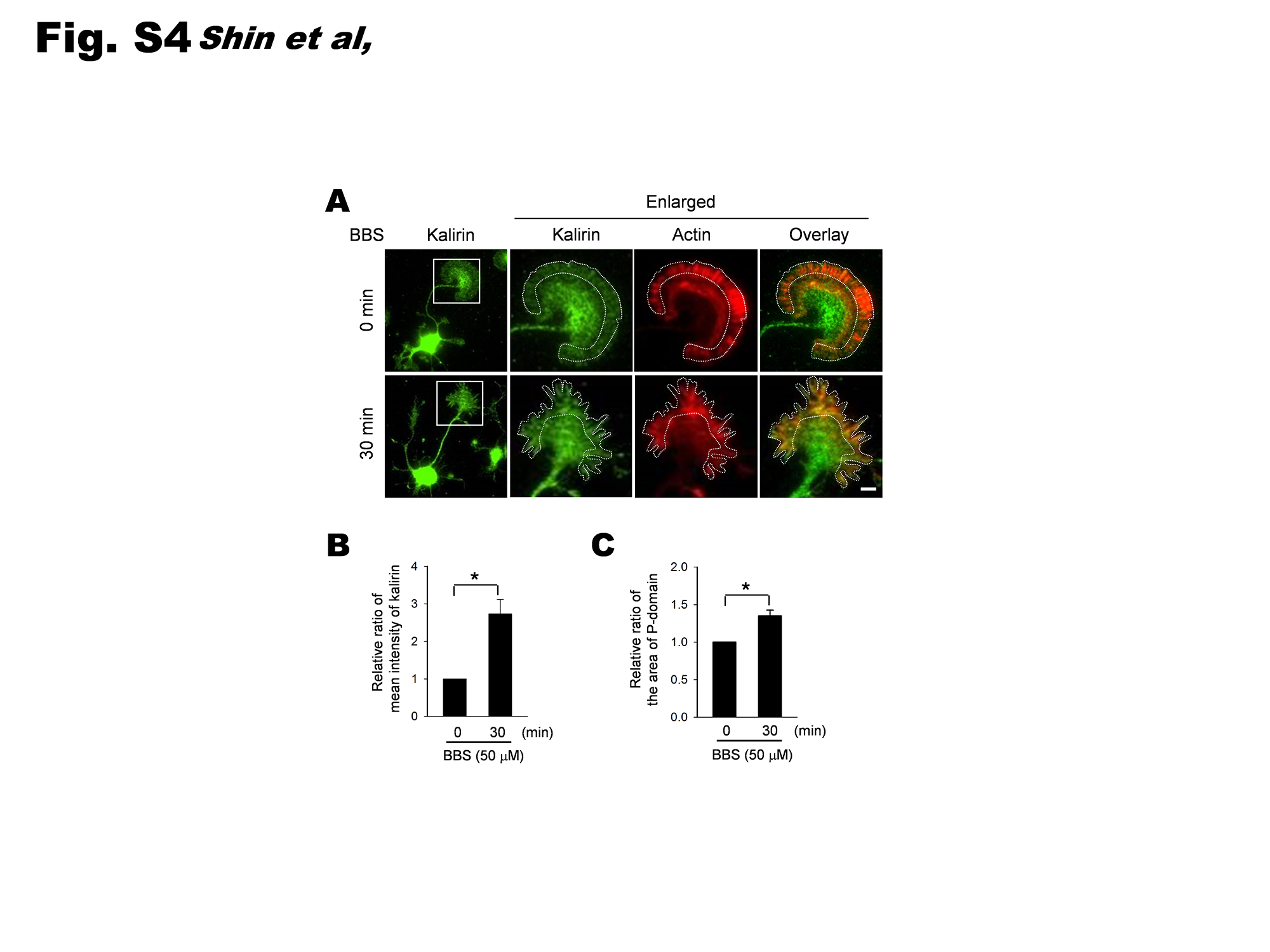

Supplement: Figure S4 — Blebbistatin alters localization of kalirin. A. HP neurons were incubated with 50 µM BBS for the indicated times and then double-stained with TRITC-labeled phalloidin for actin (red) and anti-kalirin antibody (green). The dotted lines indicate the peripheral actin–positive area. Scale bar, 10 µm. B. Quantification of kalirin localization in the peripheral actin–positive area. Mean fluorescence intensity of kalirin after BBS treatment was expressed as a relative ratio of pA'/pA. The mean intensity for pA in the BBS-untreated growth cones was set to 1. C. Quantification of the peripheral actin–positive area. The size of the peripheral actin–positive area after BBS treatment was expressed as a relative ratio of pA'/pA. The size for pA in the BBS-untreated growth cones were set to 1. The number of cells examined was 22 (for time 0) or 25 (for 30 min). Error bars are ± SD. *, P<0.05. (TIF) [file pone.0095212.s004.tif]

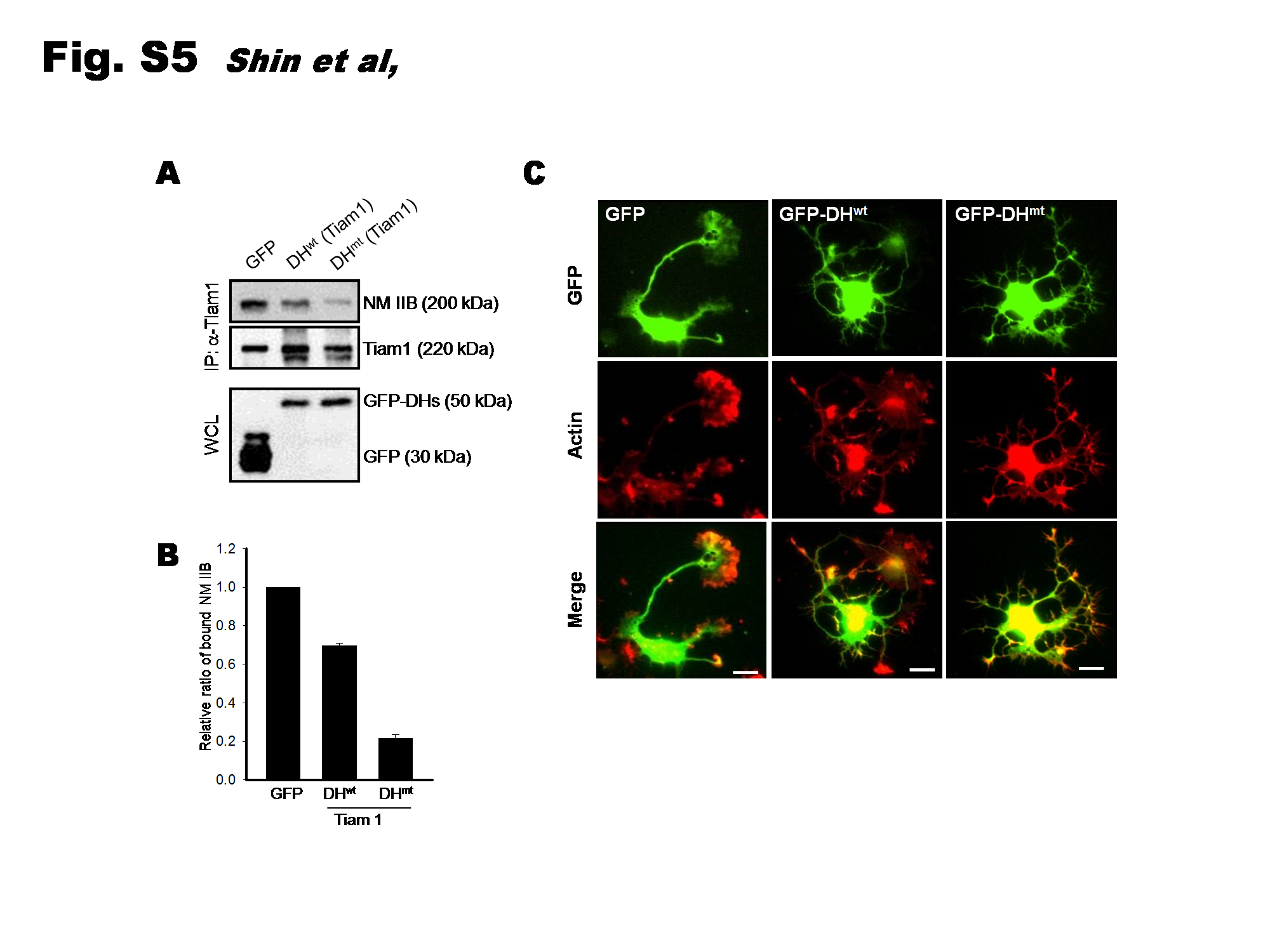

Supplement: Figure S5 — The effect of overexpression of the Tiam1 DH domain on growth cone formation and neurite branching. A. PC12 cells were transfected with plasmids encoding GFP (control), GFP-Tiam1 DHwt, or GFP-Tiam1 DHmt domain. Lysates were immunoprecipitated with anti-Tiam1 antibody, and immunoprecipitates were probed for NM IIB or Tiam1 (top). To assess the expression of transfected genes, immunoblotting for GFP was performed (bottom). The data are representative of three independent experiments. B. Quantitative analysis of immunoblots from three independent experiments shown in A. C. Cultured HP neurons were transfected with plasmids encoding GFP (control), GFP-DHwt or GFP-DHmt domain. Cells were stained for actin (red), and expression of transfected GFP constructs (green) was examined by fluorescence microscopy. Scale bar, 10 µm. (TIF) [file pone.0095212.s005.tif]

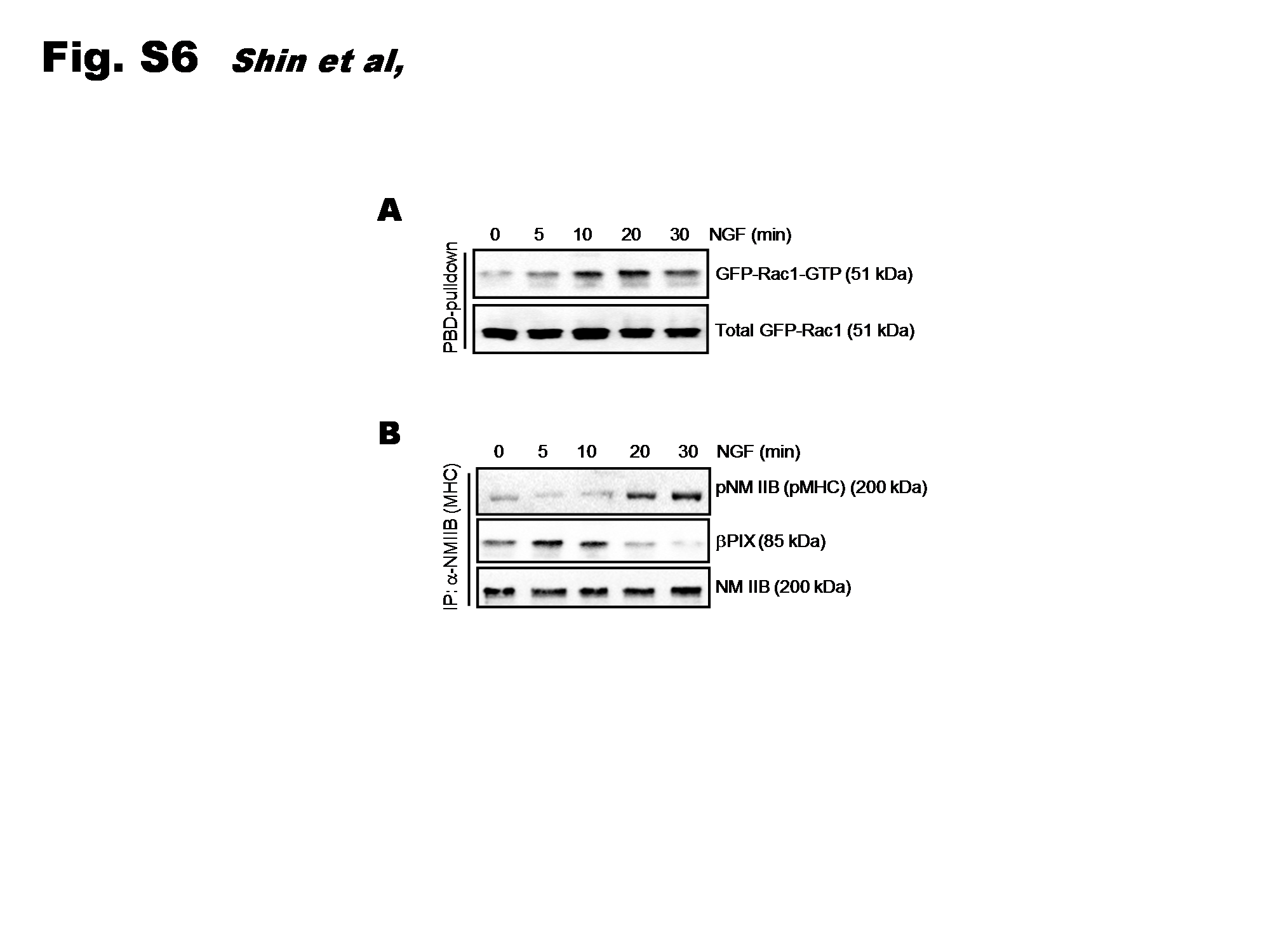

Supplement: Figure S6 — NGF stimulates dissociation of the NMII–GEF complex through Rac1 activation. A. PC12 cells were transfected with GFP-tagged Rac1 and stimulated with 100 ng/ml NGF for the indicated times. A GST-PBD pulldown assay was performed to measure Rac1 activation. B. NGF-stimulated lysates were immunoprecipitated with anti-NM IIB antibody, and immunoprecipitates were immunoblotted with anti-phospho-threonine antibody for pNM IIB, total NM IIB or βPIX antibodies. The data are representative of three independent experiments. (TIF) [file pone.0095212.s006.tif]
